# Diagnostic criteria for acute headache attributed to ischemic stroke and for sentinel headache before ischemic stroke

**DOI:** 10.1186/s10194-021-01372-x

**Published:** 2022-01-20

**Authors:** Elena R. Lebedeva, Anton V. Ushenin, Natalia M. Gurary, Denis V. Gilev, Jes Olesen

**Affiliations:** 1 Department of Neurology, the Ural State Medical University, Yekaterinburg, Russia; 2 International Headache Center “Europe-Asia”, Yekaterinburg, Russia; 3 The Urals State Medical University, Repina 3, 620028 Yekaterinburg, Russia; 4 Medical Union “New Hospital”, Yekaterinburg, Russia; 5 Department of Economics, the Ural Federal University, Yekaterinburg, Russia; 6 DanishHeadache Center, Department of Neurology, Rigshospitalet-Glostrup, University of Copenhagen, Copenhagen, Denmark

**Keywords:** Ischemic stroke, Headache, Secondary headache, Headache attributed to ischemic stroke, Sentinel headache, Headache in stroke, Diagnostic criteria

## Abstract

**Background:**

Defining the relationship between a headache and stroke is essential. The current diagnostic criteria of the ICHD-3 for acute headache attributed to ischemic stroke are based primarily on the opinion of experts rather than on published clinical evidence based on extensive case-control studies in patients with first-ever stroke. Diagnostic criteria for sentinel headache before ischemic stroke do not exist. The present study aimed to develop explicit diagnostic criteria for headache attributed to ischemic stroke and for sentinel headache.

**Methods:**

This prospective case-control study included 550 patients (mean age 63.1, 54% males) with first-ever ischemic stroke and 192 control patients (mean age 58.7, 36% males) admitted to the emergency room without any acute neurological deficits or severe disorders. Standardized semi-structured interview forms were used to evaluate past and present headaches during face-to-face interviews by a neurologist on admission to the emergency room in both groups of patients. All headaches were diagnosed according to the ICHD-3. We tabulated the onset of different headaches before a first-ever ischemic stroke and at the time of onset of stroke. We divided them into three groups: a new type of headache, the previous headache with altered characteristics and previous unaltered headaches. The same was done for headaches in control patients within one week before admission to the hospital and at the time of entry. These data were used to create and test diagnostic criteria for acute headache attributed to stroke and sentinel headache.

**Results:**

Our previous studies showed that headache at onset of ischemic stroke was present in 82 (14.9%) of 550 patients, and 81 (14.7%) patients had sentinel headache within the last week before a stroke. Only 60% of the headaches at stroke onset fulfilled the diagnostic criteria of ICHD-3. Therefore, we proposed alternative criteria with a sensitivity of 100% and specificity of 97%. Besides, we developed diagnostic criteria for sentinel headache for the first time with a specificity of 98% and a sensitivity of 100%.

**Conclusions:**

We suggest alternative diagnostic criteria for acute headache attributed to ischemic stroke and new diagnostic criteria for sentinel headache with high sensitivity and specificity.

## Introduction

Defining the relationship between a headache and stroke is essential for correct diagnosis, optimal and timely treatment, and prevention of severe neurological complications. However, headache is a widespread symptom. Migraine has a prevalence of 15% and tension-type headache prevalence 75% in the general population [1, 2]. When people get a headache associated with a stroke, it is therefore difficult to decide if it is caused by the stroke or just one of the usual headaches that randomly occur at the time of the stroke. The closer the headache is to stroke onset, the smaller the chance of a usual headache to happen just at that time. Likewise, if the headache is different from regular or has significantly altered characteristics, the likelihood of a causal relationship increases. A large prospective study with a simultaneous control group is the best way to ascertain causality. Headaches that occur in the stroke group and not in the control group are more likely to be associated with the stroke.

In previous studies, we studied sentinel headache before ischemic stroke and also headache at onset of first-ever ischemic stroke [3, 4]. However, the current diagnostic criteria for acute headache attributed to ischemic stroke are based primarily on the opinion of experts rather than on published clinical evidence based on extensive case-control studies of these headaches in patients with first-ever ischemic stroke. The diagnostic criteria might therefore need revision. Besides, diagnostic criteria for sentinel headache before ischemic stroke do not exist and could perhaps help predict and prevent a stroke. The present study aims to develop explicit diagnostic criteria for headache attributed to ischemic stroke and sentinel headache. For this, we used data from the large Yekaterinburg controlled study of headache in ischemic stroke that included a sizeable simultaneous control group.

## Materials and methods

### Study groups

This prospective case-control study was carried out between September 2012 and September 2015. The clinical part of the study was located at the stroke unit of city hospital “New Hospital”, and follow-up of patients was continued at the International headache centre “Europe-Asia” in Yekaterinburg. The study included: patients with first-ever ischemic stroke and simultaneous controls. We previously described material and methods in detail [3, 4]. Inclusion criteria for the patients with ischemic stroke: (1) 18 years and older with a first-ever ischemic stroke; (2) presence of new infarction on magnetic resonance imaging (MRI) with diffusion-weighted imaging (DWI), or computed tomography (CT); (3) the patient was examined by a neurologist on admission to the emergency room or within the first day after admission; (4) the patient agreed to be interviewed and follow-up within one year of the initial examination for the evaluation of headache after stroke for future study of persistent headache after stroke.

Exclusion criteria for the stroke group: (1) pronounced cognitive deficit, mental or speech impairments that would impede the interview; (2) a history of previous stroke/transient ischemic attacks, subarachnoid haemorrhage, unruptured cerebral artery aneurysm, intracerebral haemorrhage, brain tumour, any brain surgery, multiple sclerosis, epilepsy, encephalitis, meningitis and other severe neurological or somatic diseases; (3) patient could not give a clear description of previous and current headaches; (4) refusal to participate in the study for any reason.

As a control group, we used patients admitted to the city hospital “New Hospital” without acute neurological deficit or severe neurological or somatic disorder. The criteria for exclusion were: memory and speech disorders, history of stroke or transient ischemic attack or cerebral haemorrhage, arterial dissection, temporal arteritis and other diseases of the vessels of the brain and neck, any history of brain surgery, serious somatic pathology (cancer, decompensation of diabetes mellitus, hepatitis/cirrhosis, etc.) high blood pressure more than 180/100 mm Hg, infections, the presence of a brain tumour, traumatic brain injury and the trauma of the cervical spine in history, epilepsy, multiple sclerosis, dementia.

### Evaluation

This study was done prospectively. Standardized semi-structured interview forms were used for evaluation of past and present headaches during face-to-face interviews by a neurologist on admission to the emergency room or within the first day after admission in both in patients with stroke and in controls.

To analyze all characteristics of headache at stroke onset and to test existing diagnostic criteria, we referenced the exact time of the first stroke symptom. All headaches at the time of stroke and withing seven days before stroke were compared with pre-existing headaches during the previous year. When changes in the characteristics of a previous headache were found, they were specified. Headaches were classified as a new type, a previous headache with altered characteristics or as a previous headache without altered characteristics. The time and the day of the disappearance of headache were determined when short-lasting or else at a follow-up three months after stroke.

### Definitions and diagnostic criteria

All headaches were diagnosed according to the International Classification of Headache Disorders 3rd edition (ICHD-3) [5]. We defined sentinel headache as a new type of headache or a previous kind of headache with altered characteristics (severe intensity, increased frequency, absence of effect of drugs) which arose within seven days before stroke [3].

Headaches at the time of stroke were defined as within 24 h after stroke onset [4]. A new type of headache was defined as a headache that arose for the first time within 24 h after stroke onset or before the stroke. If headache had changed characteristics (headache became more severe, longer-lasting, more frequent, new accompanying symptoms developed or analgesics became ineffective for pain relief) it was defined as a headache with altered characteristics. If a pre-existing headache occurred unaltered, the headache was defined as a usual headache without changes.

### Methodology for developing new diagnostic criteria for headache attributed to stroke and sentinel headache before stroke

There is no gold standard that shows whether or not a headache is attributed to stroke. We, therefore, first tabulated the onset of different headaches before a first-ever stroke and at the time of onset of stroke. We divided them into three groups: a new type of headache, the previous headache with altered characteristics and previous unaltered headaches. The same was done for headaches in control patients within one week before admission to the hospital and at the time of entry. By preliminary analysis, it was clear that extremely few patients in the control group had a new headache or a headache with altered characteristics within seven days before admission and during 24 h after admission, while previous headaches without altered characteristics were equally prevalent in stroke patients and controls. Therefore, we defined that the first two types of headaches were causally related to stroke and used this to test existing criteria and develop new diagnostic criteria. These data were used to create and test diagnostic criteria for acute headache attributed to stroke and sentinel headache.

The use of sub-criterion C2 is redundant since all headaches occurred within 24 h. Besides, this criterion is difficult to determine. We suggest deleting sub-criterion C2 (Table 1) since all headaches occurred within 24 h.

**Table 1:** Diagnostic criteria for headache attributed to ischemic stroke of the ICHD-3

A. Any new headache fulfilling criteria C and D.
B. Acute ischaemic stroke has been diagnosed.
C. Evidence of causation demonstrated by either or both of the following:
1. headache has developed in very close temporal relation to other symptoms and/or clinical signs of ischaemic stroke or has led to the diagnosis of ischaemic stroke
2. headache has significantly improved in parallel with stabilization or improvement of other symptoms or clinical or radiological signs of ischaemic stroke
D. Either of the following:
1. headache has resolved within 3 months
2. headache has not yet resolved but 3 months have not yet passed
E. Not better accounted for by another ICHD-3 diagnosis.

We also tabulated the onset of different types of headaches before the stroke and before the admission of controls. These data were used to develop and test diagnostic criteria for sentinel headache. The aim was that the criteria should identify headaches that occurred in the stroke patients within a week before an ischemic stroke but not before the admission of the control patients.

### Statistical analysis

All patients with stroke included in this study were submitted to testing of the criteria for headache attributed to ischemic stroke. The number of patients fulfilling each criterion was recorded. The number fulfilling each sub-criterion in the stroke group was compared to the number in the control group. Finally, the number fulfilling the full revised criteria was compared to the control group.

Statistical analyses were performed with Stata (ver.14.0) and Microsoft Excel (2014). The basic comparisons were between patients with ischemic stroke and controls and patients with and without headaches before and at stroke onset. Univariate analyses were performed to calculate crude odds ratios (OR) with 95% confidence intervals (CI). The sensitivity of criteria was calculated as number fulfilling criteria x 100 / number with a new headache or a headache with altered characteristics in the total material. Specificity was calculated as number fulfilling criteria in total material minus number fulfilling criteria in control groups / total number with new headaches or headaches with altered characteristics in stroke and control group.

## Results

We examined in total 2995 patients with ischemic stroke and 225 control patients. Most stroke patients were, however, excluded from the study due to exclusion criteria. Five hundred fifty patients with first-ever ischemic stroke and 192 control patients were included.

Stroke in the anterior circulation system was found in 456 patients (82.9%), 94 patients (17.1%) had a stroke in the posterior circulation system. All patients had stroke onset within 24 h before admission to the hospital. Most of the patients (75%) had stroke onset 12-24 h before admission, 10% within 4-12 h, 15% ≤ 3 h before admission to the hospital.

Control patients admitted to the hospital mainly because of acute pain syndromes of different localizations: “lumbago” (*n* = 99), “pancreatitis” (*n* = 62), “peptic ulcer” (*n* = 7), “irritable bowel syndrome” (*n* = 2), “osteoarthritis” (*n* = 5). Other patients had diagnoses such as “tick bite” (*n* = 14), “paroxysmal benign positional vertigo” (*n* = 2), “allergic reaction” (*n* = 1).

The mean age of patients with stroke and controls did not differ significantly: 63.0 and 58.7 but females were overrepresented in the control group (64% and 46% comparatively).

### Present and new diagnostic criteria for acute headache attributed to ischemic stroke

The timing of headache to stroke and admission of control patients is presented in Table 2. These data allowed testing the current diagnostic criteria for Headache Attributed to ischemic stroke of the ICHD-3 (Table 1).

**Table 2:** The time of development of headache on the day of the onset of first-ever ischemic stroke or on the day of admission of controls (in parenthesis)

Type of headache	The time of the development of headache									
	**Simultaneously** with the onset of stroke (admission of controls)	**During 15 min after** the onset of stroke (after admission of controls)	**15-30 min after** the onset of stroke (after admission of controls)	**30-45 min after** the onset of stroke (after admission of controls)	**45-60 min after** the onset of stroke (after admission of controls)	**1-2 h after** the onset of stroke (after admission of controls)	**2-3 h after** the onset of stroke (after admission of controls)	**3-4 h after** the onset of stroke (after admission of controls)	**4-24 h after** the onset of stroke (after admission of controls)	**Total**
**A new type of headache**	46 (0)	0 (0)	0 (0)	0(0)	0 (0)	0(0)	0 (0)	0 (0)	0 (0)	46(0)
**Headache with altered characteristic**	14 (0)	8 (0)	1 (0)	0 (0)	1 (0)	0(0)	4 (0)	2 (2)	0 (0)	30(2)
**The previous headache without changes**	4 (0)	0 (0)	0 (0)	0 (2)	0 (0)	0(1)	2 (0)	0 (0)	0 (6)	6 (9)

The criteria state that only new headaches are attributed to stroke. However, at the onset of first-ever ischemic stroke, only 56% of patients with headache had a new type of headache (mainly migraine-like), and 36% had a headache with altered characteristics (mainly tension-type-like headache). No controls had a new type of headache, and only two control patients had a headache with altered characteristics at admission to the hospital (Table 2). Therefore, we suggest including headaches with altered characteristics in the revised criteria.

It is unclear what “very close temporal relation” means. We suggest “24 hours of other symptoms and/or clinical signs of ischaemic stroke”. All headaches of a new type and 47% of headaches with altered characteristics developed within one hour of stroke onset, and in the remaining patients, headaches with altered characteristics arose within 24 h after stroke.

The phrase of subcriterion C1 “headache has led to the diagnosis of ischaemic stroke” does not make sense in these criteria since such headaches are sentinel headaches that should have separate diagnostic criteria.

The sensitivity of the current diagnostic criteria for acute headache attributed to stroke is only 46 × 100/76=60%. Therefore, we suggest the following alternative criteria for acute headache attributed to ischemic stroke (Table 3).

**Table 3:** Alternative diagnostic criteria for acute headache attributed to ischemic stroke

A. Any headache fulfilling criteria B and C.
B. Acute ischaemic stroke has been diagnosed.
C. Evidence of causation demonstrated by the following:
1. a new headache has developed within 24 hours of other symptoms and/or clinical signs of ischaemic stroke
2. a previous headache with altered characteristics has developed within 24 hours of other symptoms and/or clinical signs of ischemic stroke
D. Either of the following:
1. headache has resolved within three months
2. headache has not yet resolved but three months have not yet passed
E. Not better accounted for by another ICHD-3 diagnosis.

Table 4 shows the fulfilment of ICHD-3 diagnostic subcriteria and proposed subcriteria for headache attributed to ischemic stroke. The sensitivity of these proposed criteria was 78х100/78=100%, and the specificity was (78-2)х100/78=97%.

**Table 4:** Fulfilment of ICHD-3 diagnostic subcriteria and proposed subcriteria for headache attributed to ischemic stroke

Diagnostic criteria for headache attributed to ischemic stroke	Fulfilment of diagnostic criteria (number of patients with stroke)
A. **ICHD-3 diagnostic criteria**	
A. Any new headache fulfilling criteria C and D	46
B. Acute ischaemic stroke has been diagnosed	46
C. Evidence of causation demonstrated by either of the following:	
C1. Headache has developed in very close temporal relation to other symptoms and/or clinical signs of ischaemic stroke, or has led to the diagnosis of ischaemic stroke	46
C2. Headache has significantly improved in parallel with stabilization or improvement of other symptoms or clinical or radiological signs of ischaemic stroke	25
D. Either of the following:	
D1. Headache has resolved within 3 months	25
D2. Headache has not yet resolved but 3 months have not yet passed	21
Total fulfilling ICHD-3 criteria	46
B. **Proposed alternative diagnostic criteria**	
A. Any headache fulfilling B and C	76
B. Acute ischaemic stroke has been diagnosed	76
C. Evidence of causation demonstrated by the following:	
C1 A new headache has developed within 24 h of other symptoms and/or clinical signs of ischaemic stroke of other symptoms and/or clinical signs of ischaemic stroke	46
C2 A headache with altered characteristics has developed within 24 h of other symptoms and/or clinical signs of ischaemic stroke of other symptoms and/or clinical signs of ischaemic stroke	30
D. Either of the following:	
D1. Headache has resolved within 3 months	25
D2. Headache has not yet resolved but 3 months have not yet passed	51
Total fulfilling proposed criteria	76

Table 5 shows headaches occurring 2-7 days after stroke. We had no controls for this time period. In total, there were only 4 with a new headache or a headache with altered characteristics. They may be attributed to stroke, but our data do not allow us to conclude that. Even if they were caused by stroke, it would hardly be worth including them in diagnostic criteria as it would impair specificity.

**Table 5:** The onset of headache during seven days after first-ever ischemic stroke^*^

Type of headache	The day of the development of headache after stroke					
	On the second day after stroke	On the third day after stroke	On the fourth day after stroke	On the fifth day after stroke	On the sixth day after stroke	On the seventh day after stroke
**A new type of headache**	1	1	0	0	0	0
**Headache with altered characteristic**	0	1	0	1	0	1
**The previous headache without changes**	1	0	0	1	0	0
**Total**	2	2	0	2	0	1

*No control data available

### Diagnostic criteria for sentinel headache

The ICHD-3 does not include diagnostic criteria for sentinel headache before an ischemic stroke. Our previous study [3] characterized the prevalence and clinical characteristics of sentinel headache before ischemic stroke in 550 patients with first-ever ischemic stroke. Here we propose explicit diagnostic criteria for sentinel headache. Totally 94 patients had headaches within the last week before a stroke and 12 in the control group. 81(14.7%) had a new headache or a headache with altered characteristics and two controls (1.0%). Therefore, these headaches are true sentinel headaches. It was most often a migraine-like headache, rarely a thunderclap headache. The duration of sentinel headache in most patients (69.1%) was within 24 h of onset, in 19.7% up to 48 h, in 8.6% up to four days, in 2.5% up to three weeks. In 30.9% of the patients, sentinel headache continued during the onset of stroke [3]. Table 6 shows the time and the day of development of sentinel headache. According to these data, we elaborated criteria for sentinel headache (Table 7).

**Table 6:** The onset of sentinel headache before stroke in relation to the time of stroke or headaches during seven days before admission of the control group

The time/day of development of headache	Hours before stroke/admission of controls	1 day before stroke/admission of controls	2 days before stroke/admission of controls	3 days before stroke/admission of controls	4 days before stroke/admission of controls	5 days before stroke/admission of controls	6 days before stroke/admission of controls	7 days before stroke/admission of controls	Total
**A new type of headache (***n***=27)**	10 (0)	5 (0)	8 (0)	3 (0)	1 (0)	0 (0)	0 (0)	0 (0)	27(0)
**Headache with altered characteristic (***n***=54)**	13 (0)	6 (1)	7 (1)	1 (0)	4 (0)	7 (0)	6 (0)	10 (0)	54(2)
**The previous headache without changes (***n***=13)**	2 (1)	5 (3)	3 (2)	2 (2)	1 (2)	0 (0)	0 (0)	0 (0)	13(10)

**Table 7:** Diagnostic criteria for sentinel headache before ischemic stroke

A. Any headache fulfilling criteria B and C
B. Acute ischaemic stroke has been diagnosed
C. Evidence of causation demonstrated by least one of the following:
1. a new headache never experienced before developed within one week before ischaemic stroke
2. a previous headache with altered characteristics (severe intensity, increased frequency, prolonged duration, development of new accompanying symptoms, absence of effect of drugs for pain relief) developed within one week before ischaemic stroke
D. Not better accounted for by another ICHD-3 diagnosis.

Table 8 shows the fulfilment of the proposed subcriteria for sentinel headache. The specificity of these criteria was (83-2)x100/83=98%. The sensitivity was (83-0)x100/83=100%.

**Table 8:** Fulfilment of proposed subcriteria for sentinel headache

Diagnostic criteria	Fulfilment of diagnostic criteria (number of patients with stroke)
**Proposed diagnostic criteria for sentinel headache**	
A. Any headache fulfilling criteria B and C	81
B. Acute ischaemic stroke has been diagnosed	81
C. Evidence of causation demonstrated by either of the following:	
C1. A new headache that had never been experienced before has developed within one week before other symptoms	27
C2. A previous headache with altered characteristics has developed within one week before other symptoms	54
Total fulfilling proposed criteria	81

## Discussion

The existing diagnostic criteria for acute headache attributed to ischemic stroke follow the general diagnostic criteria for secondary headache [5]. Our data show, however, that one size does not fit all. The sensitivity of the existing criteria was far too low because many headaches after stroke not seen in the control group did not fulfil ICHD-3 criteria. We propose alternative diagnostic criteria that include a new type of headache and a previous type of headache with altered characteristics. The proposed criteria had much higher sensitivity and preserved specificity because extremely few headaches in the control group fulfil the criteria.

Sentinel headache was described more than 20 years ago by Gorelick PB et al. [6]. Sentinel headache is considered as a signal of the impending rupture of an aneurysm [7]. Since we studied symptoms before a rupture of intracranial aneurysms and observed such headaches [8], we decided to assess the presence of sentinel headache before ischemic stroke and transient ischemic attacks (TIA) [4, 9]. According to our previous studies, 18.3% of the patients with TIA had sentinel headache within the last week before TIA [9], and 14.7% had sentinel headache before ischemic stroke [4]. We found that attacks of arrhythmia before a stroke and a history of atrial fibrillation were associated with this headache in stroke patients [4]. These data suggest that emboli can play an essential role in the development of this headache [4]. However, sentinel headache may be an alarming symptom in many other disorders (dissections, cerebral venous sinus thrombosis, etc.). Therefore, it is necessary to study further this headache using diagnostic criteria. However, they have not been developed before. First of all, it was impossible in most studies because they were simple descriptions of headaches using stroke registry data. Besides, a sizeable concurrent control group is necessary to reveal a casual association between headache and stroke. A detailed interview about previous headaches and headaches within the last seven days before the stroke is also essential. It turned out that sentinel headaches were relatively frequent and that explicit diagnostic criteria separated them very efficiently from previous headaches.

### Developing and testing explicit diagnostic criteria for secondary headaches

The main problem in developing explicit diagnostic criteria for secondary headaches is the very high prevalence of migraine (15%) and tension-type headache (75%). Most people have experienced primary headaches before a possible secondary cause of headache appears. Is the headache then causally related to this disorder, or is it a usual headache, a migraine, or a tension-type headache? There are only a few ways in which headaches can be expressed. In the first edition of the International classification of headache disorders (ICHD-1), a list was given of the different types of headaches: migraine-like, tension-type-like, cluster-like, thunderclap-like or orthostatic. In the present study, we encountered primarily migraine-like headaches and tension type-like headaches in association with stroke. The best method to decide causality is to compare to a simultaneous control group as done in the present study. There was an apparent separation between stroke patients and controls in the present study regarding a new type of headache or a usual headache with altered characteristics. Therefore, such headaches were attributable to the stroke, and a similar clear separation was seen concerning sentinel headache one week before the stroke. It is not likely that similar studies of other secondary causes will result in similarly precise results. The slower the onset of the secondary cause (such as a benign brain tumour), the more difficult it is to find a suitable control group.

There were no explicit pre-defined criteria for altered characteristics in the present study, although there was a list of such characteristics used by the interviewer. This opens for bias as the interviewers could easily interpret headaches to have altered characteristics in stroke patients but not in control patients. But that seems not to have been the case since the prevalence of usual headaches with unaltered characteristics was the same in stroke patients and the control patients. Any misinterpretation would have resulted in many usual headaches without altered characteristics being wrongly allocated to the group of headaches with altered characteristics. Still, in that case, there would be fewer usual headaches in the stroke group. Since the group of headaches with altered characteristics is so large, it suggests that the methodology of future studies of secondary headaches should include pre-determined criteria for altered characteristics. Furthermore, it seems advisable that the general criteria for secondary headaches be revised to include the distinction between previous headaches with altered characteristics and previous headaches without altered characteristics.

### Strengths and weaknesses of the present study

The present study capitalizes on the huge Yekaterinburg stroke study, which included prospective, systematic interviews in the acute phase of stroke screening of almost 3000 patients, including 550 and 192 control patients. This made it possible to test existing criteria and develop new, more sensitive and still specific diagnostic criteria. However, it is a problem to first use such material to develop criteria and later test for sensitivity and specificity in the same patient material. It resulted in 100% sensitivity and 98% specificity, which may be somewhat overestimated. Prospective testing of our criteria in novel cohorts of patients and controls is advisable in future studies.

Patients with impaired consciousness, cognitive impairment and speech disturbances were excluded from the study since it is impossible to obtain detailed information about previous history of headache and current headache in such cases. 76.5% of patients had National Institutes of Health Stroke Scale score less than 8. It doesn’t allow to generalize our findings to the overall population of ischemic stroke. However, results of previous studies showed that headache at stroke onset was associated with good neurological status and good outcome [4, 10].

Men prevailed among patients with stroke and women in the control group because they were more frequently admitted to the emergency room. This would lead to a higher prevalence of headaches in the females of controls; however, it was found that headaches prevailed in patients with stroke.

The control group of 192 patients was mainly composed of acute pain syndromes such as lumbago (*n*=99) and pancreatitis (*n*=62). These disorders could lead to an increased occurrence of headaches, even medication overuse headaches, since some patients (*n*=5) frequently use pain killer drugs for their pathologies. However, it did not happen. These facts confirm the role of stroke in the development of headaches.

## Conclusions

The existing diagnostic criteria for acute headache attributed to ischemic stroke are based primarily on the opinion of experts rather than on published clinical evidence based on extensive case-control studies of these headaches in patients with first-ever ischemic stroke. They are too insensitive since only 60% of acute headaches at stroke onset fulfilled the diagnostic criteria of ICHD-3. Explicit diagnostic criteria for headache attributed to ischemic stroke were developed in the present prospective case-control study. They have high sensitivity and specificity.

Diagnostic criteria for sentinel headache were developed for the first time. They are sensitive and specific. Both sets of criteria should be used in future editions of the International Classification of Headache Disorders.

## Data Availability

The datasets used and analyzed during the current study are available from the corresponding author on reasonable request.

## Abbreviations

CT: Computed Tomography
MRI: magnetic resonance imaging
DWI: Diffusion-weighted imaging
TIA: transient ischemic attacks
OR: odds ratio
CI: confidence interval
ICHD-3: International Classification of Headache Disorders
